# A systematic survey of randomised trials that stopped early for reasons of futility

**DOI:** 10.1186/s12874-020-0899-1

**Published:** 2020-01-16

**Authors:** S. D. Walter, H. Han, G. H. Guyatt, D. Bassler, N. Bhatnagar, V. Gloy, S. Schandelmaier, M. Briel

**Affiliations:** 1 Department of Health Research Methods, Evidence, and Impact, McMaster University, Hamilton, Ontario L8N 3Z5 Canada; 2 Mount Sinai Hospital, Toronto, Canada; 3 Department of Neonatology, University Hospital Zurich and University of Zurich, Zurich, Switzerland; 4 Basel Institute for Clinical Epidemiology and Biostatistics, Department of Clinical Research, University of Basel and University Hospital Basel, Basel, Switzerland

**Keywords:** Clinical trials, interim analysis, early stopping rules, treatment effect size, bias

## Abstract

**Background:**

Randomised trial protocols may incorporate interim analyses, with the potential to stop the study for futility if early data show insufficient promise of a treatment benefit. Previously, we have shown that this approach will theoretically lead to mis-estimation of the treatment effect. We now wished to ascertain the importance of this phenomenon in practice.

**Methods:**

We reviewed the methods and results in a set of trials that had stopped for futility, identified through an extensive literature search. We recorded clinical areas, interventions, study design, outcomes, trial setting, sponsorship, planned and actual treatment effects, sample sizes; power; and if there was a data safety monitoring board, or a published protocol. We identified: if interim analyses were pre-specified, and how many analyses actually occurred; what pre-specified criteria might define futility; if a futility analysis formed the basis for stopping; who made the decision to stop; and the conditional power of each study, i.e. the probability of statistically significant results if the study were to continue to its complete sample size.

**Results:**

We identified 52 eligible trials, covering many clinical areas. Most trials had multiple centres, tested drugs, and 40% were industry sponsored. There were 75% where at least one interim analysis was planned a priori; a majority had only one interim analysis, typically with about half the target total sample size. A majority of trials did not pre-define a stopping rule, and a variety of reasons were given for stopping. Few studies calculated and reported low conditional power to justify the early stop. When conditional power could be calculated, it was typically low, especially under the current trend hypothesis. However, under the original design hypothesis, a few studies had relatively high conditional power. Data collection often continued after the interim analysis.

**Conclusions:**

Although other factors will typically be involved, we conclude that, from the perspective of conditional power, stopping early for futility was probably reasonable in most cases, but documentation of the basis for stopping was often missing or vague. Interpretation of truncated trials would be enhanced by improved reporting of stopping protocols, and of their actual execution.

## Background

Clinical trial investigators often strive to minimise the total number of patients who participate in their study, arrive at a rapid decision about treatment effects, and lower study costs. Carrying out early analyses of the interim data, with the possibility of stopping the study early if sufficiently convincing results are found, is therefore appealing. A variety of stopping rules have been proposed for trial protocols when interim analyses are planned, giving investigators a formal way to evaluate the interim results, and providing statistical guidance to decide if the study should indeed be stopped.

One type of rule concerns possible stopping for *benefit*, if the early data provide strong evidence of an important treatment effect (usually in favour of the experimental treatment). Most such rules try to control overall type I (or *α*) statistical error rates during a series of actual or planned interim analyses. Specific stopping rules can be characterised by their strategy of *α*-spending, or equivalently how the decision thresholds on the magnitude of treatment effect are defined at the time of each interim analysis. Some rules use a constant *α* for all the interim analyses, while others require stronger statistical significance (by using a smaller *α*-value) at early analyses when limited data are available, and correspondingly less stringent criteria (larger *α*) for later analyses.

A second type of rule concerns possible stopping for *futility*, which may occur when the early data suggest that an important treatment effect is unlikely to be found, even if the study were to continue to its full planned sample size. Investigators may stop the study if an interim analysis shows little difference between the experimental and control groups, or even that the control treatment appears to be superior. In these cases, the decision about early stopping can be informed by a calculation of conditional power, defined as the probability of obtaining a statistically significant result if the study is allowed to continue to its planned completion. Conditional power can be evaluated after making alternative assumptions about the future data, including two common choices: *i*) the current trend observed in the interim data will continue; or *ii*) the original treatment effect size proposed in the study design is retained and still assumed to be correct. Other possibilities are to assume (optimistically) the upper limit of an interim confidence interval for the future treatment effect, for example at the 80% or 95% confidence level, or (pessimistically) to adopt the null hypothesis of no treatment effect.

In our previous work [1], we developed the theory of how a futility stopping rule can affect the estimated treatment effect, together with an illustrative example. Our results showed that the impact of a stopping rule depends on several factors, including the true underlying treatment effect, the magnitude of treatment effect that the trial is designed to detect, study power, the number of planned interim analyses, and what assumption is made about future data to be observed after an interim analysis. As might be anticipated intuitively, we showed that if a study actually stops for reasons of futility, its treatment effect will tend to be under-estimated, possibly substantially so. Less intuitively, we showed how in studies with a stopping rule in place, but which do not actually stop at an interim analysis, there is some over-estimation of the true effect. Finally, we demonstrated that there is an overall negative bias in studies with a futility stopping rule in place. However, because there is only a small probability of stopping early in many practical situations, the overall bias is often small, and the more serious concern is therefore the potential for substantial under-estimation of the treatment effect in studies that do actually stop for futility.

From a survey of 894 randomised controlled trial protocols enrolling patients in the DISCOntinuation of randomised controlled trials (DISCO) study [2], it was found that 289 (32%) had pre-specified the possibility of interim analyses, and 153 (17%) had one or more stopping rules. Specifically, 38 (4.3%) protocols had specified an interim analysis for futility, and 21 out of these 38 protocols had explicitly specified a statistical stopping rule. Overall, 46/894 trials (5.1%) were eventually discontinued for early benefit or futility, but 37 of those (80%) were not stopped based on a formal interim analysis or stopping rule.

In this paper we describe a systematic survey of the literature that identified a series of trials that apparently had stopped early for reasons of futility. We reviewed the full text of papers reporting these studies, and reviewed their protocols or trial registry entries if available, in order to document a variety of their methodological features, with a focus on aspects of the stopping rule (if any) and its implementation. Additionally, wherever possible, we calculated the conditional power that the study had to identify the clinically important treatment effect, as specified at the outset. Our objectives were to summarise the current practices being used in the decision to stop a trial for futility, to comment on the reporting of this aspect of the research, and to assess the consistency of the conditional power values with the actual decisions to truncate a study.

## Methods

### Eligibility criteria and search strategy

We included publications of randomised controlled trials (RCTs) that (1) investigated superiority of an intervention; (2) reported early stopping of the trial in the title or abstract; and (3) explicitly reported stopping for futility in the full text. Hence, we did not consider one-arm trials, non-inferiority RCTs, or the stopping of individual trial arms while the remaining trial continued, but multi-arm trials were admissible. We systematically searched Medline and Embase using the Ovid interface for discontinued RCTs published between January 2010 (which was when the Medical Subject Heading *Early Termination of Clinical Trials* was introduced) and August 2017. The search strategy was designed with the help of an experienced research librarian (NB) and included the Medical Subject Heading and combinations of text words (see Appendix 1 for our detailed search strategy). The same search strategy was used previously for two other meta-epidemiologic studies on discontinued RCTs [3, 4]. We updated the search in August 2017. Teams of two investigators independently screened abstracts and potentially eligible full texts. They resolved disagreements about final eligibility by discussion or arbitration with a third investigator (MB). In addition, we included 18 RCTs stopped for futility that were identified in a previous retrospective cohort study on the prevalence and characteristics of discontinued RCTs (the DISCO study) [5, 6].

From the full text report of each trial, we recorded and summarised the essential details of the study condition and its clinical interventions, the study design, together with the definition of the main study outcome and its statistical type. We also noted the clinical setting, the study sponsorship, if there was a data safety and monitoring board, if there was a published protocol cited or a mentioned trial registration number; and if CONSORT guidelines were mentioned. The anticipated treatment effect on the main outcome (which we will refer to as the design effect size), as specified by the investigators, was noted. The planned and actual total sample sizes, and the study power as planned at the study outset were obtained. We identified if interim analyses had been specified a priori, and, if so, how many such analyses there had been, with their associated planned and actual sample sizes. We recorded the criteria for evaluating statistical significance of the treatment effect on the main study outcome (including 1 vs. 2*-*sided significance testing), and any pre-specified criteria for assessing futility. We determined if a formal futility analysis was reported, and if it had been the basis for truncating the study at an interim analysis, or if a different reason was given. We also noted who made the decision to stop the trial and if data collection had continued after the final interim analysis.

Whenever possible, we calculated the conditional power of the study at the time of stopping, that being the probability of obtaining a statistically significant result if the study were to continue to its original, full sample size. If studies had reported an explicit value of conditional power themselves, we retained it. For studies where conditional power was not reported by the authors, we calculated it ourselves whenever possible, based on data provided in study reports, and using both the current trend and design hypotheses. For the remaining studies, where there was insufficient information to calculate conditional power, their claims of futility could not be explicitly verified.

Conditional power at the *k*^th^ analysis is defined as the probability of rejecting the null hypothesis at the completion of the study, given the data obtained and assuming a hypothesized true treatment *θ* [7]. If the calculated value of conditional power falls below a fixed threshold, early termination for futility is recommended. Threshold values for conditional power vary depending on the context of the trial, but values in the 0.1 to 0.3 range are consistent with most common trial designs, and typical threshold values are in the range of 0.10–0.15 if the originally assumed treatment effect is correct [7–9]. We used 0.15 for the threshold conditional power in our present calculations. One-sided conditional power at the *k*th interim analysis was computed as

$$ C{P}_k\left(\theta \right)=1-\varPhi \left(\frac{c_a-{Z}_k\sqrt{t_k}-\theta \sqrt{I_K}\left(1-{t}_k\right)}{\sqrt{1-{t}_k}}\right) $$

and the two-sided conditional power at the *k*^th^ interim analysis as

$$ C{P}_K\left(\theta \right)=1-\varPhi \left(\frac{c_{\alpha }-{Z}_k\sqrt{t_k}-\theta \sqrt{I_K}\left(1-{t}_k\right)}{\sqrt{1-{t}_k}}\right)+\varPhi \left(\frac{-{c}_a-{Z}_k\sqrt{t_k}-\theta \sqrt{I_K}\left(1-{t}_k\right)}{\sqrt{1-{t}_k}}\right) $$

where *Ф*(.) is the standard normal cumulative distribution function, *c*_*α*_ *= Ф*^*− 1*^(1-*α*), *Z*_*k*_ is the test statistic calculated from the observed data at the *k*th interim analysis, *t*_*k*_ = *I*_*k*_/*I*_*K*_(0 < *t*_*k*_ ≤ *t*_*K*_ = 1) represents the proportion of the total information (often referred to as the “information time”), and *I*_*k*_ is the information at the *k*th analysis (*k* = 1,2, …, *K*), ${I}_k=\mathrm{SE}{\left({\hat{\theta}}_k\right)}^{-2}$ [7, 9, 10].

In some cases where a study feature was not reported, we felt able to make reasonable assumptions as to what it might be. For instance, some studies did not explicitly say they would be testing the treatment effect against a null value of zero, but such a value seemed plausible in the context of the other information in the study report. Some assumed values are noted as such in our results.

## Results

**Fig. 1:**
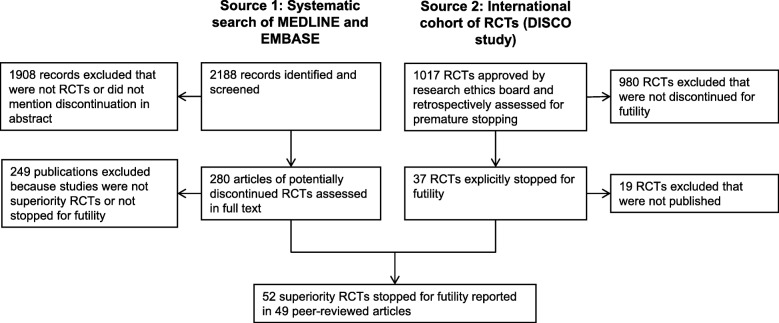
Identification of 52 randomised trials stopped for futility

Figure 1 shows how the systematic searches of Medline and EMBASE, combined with a cohort of RCTs from the DISCO project, eventually produced our final sample of 52 eligible studies; citations for these studies are provided in Additional file 4. Additional file 1 gives full details of each study on the following features: authors; clinical area and target condition; treatment and control interventions; sponsorship (industry or non-industry); if CONSORT guidelines were mentioned; if a protocol was reported, and if it was in a journal publication or in a registry only; the study design; clinical setting and geographic region; if there was a data safety and monitoring board (DSMB) mentioned; if interim analyses for futility were planned, and how many; the basis for a stopping rule, if any; and the actual number of interim analyses executed. Additional file 2 lists: the planned sample size; the type I error rate, and if 1- or 2-sided testing was used; the planned study power; the primary outcome type; the outcome variable and its null (or control) value; and the study design hypothesis in terms of expected treatment outcomes or effect sizes. Finally, Additional file 3 gives: the information percent or target sample sizes for planned interim analyses; the actual information percent at the last interim analysis; if there was a reported futility analysis, or simply a claim of futility; the basis for stopping the trial, and who made the decision to stop; the main result (or effect size), with *p*-values; if conditional power was reported or could be calculated from published details; conditional power values; and if further analyses were executed after the last planned interim analysis.

Table 1 summarises the distributions of the most important design characteristics of the sampled trials, including administrative details. Most studies used the parallel group design, and they generally occurred in multiple trial centres, either nationally or internationally. While there was a wide variety of clinical settings and therapeutic areas, a substantial majority (79%) involved a drug intervention. A placebo type of control was the most common (60%). About 40% of trials were industry-sponsored. A majority of trials had a DSMB. Only 19% of trial reports mentioned CONSORT guidelines. Protocols were available for four (8%) trials only, for an additional 22 (42%) trials we found a trial registry entry, and for 26 (50%) there was no pre-specified information available.

**Table 1:** Characteristics of 52 clinical trials that stopped for futility

Characteristics		N
Trial design	Parallel/ Factorial/ Cross-over/Cluster	50/0/0/2
Trial centres	Single	4
	Multiple, national	22
	Multiple, international	24
	Unclear	2
Setting	Outpatient/ Inpatient / Acute care	33/ 11/ 8
Clinical area	Oncology	16
	Cardiovascular research	9
	Mental health	4
	Obstetrics and gynaecology	4
	Infectious diseases	3
	Surgery	3
	Gastrointestinal	3
	Intensive care	3
	Other^a^	7
Type of intervention	Drug/ Surgical procedure/ Other	41/ 3 / 8
Type of control	Active intervention/ placebo/ no intervention	14/31/ 7
Trial sponsor	Industry/ Non-industry	21/31
Data safety and monitoring board	Yes/No	36/ 16
CONSORT mentioned	Yes/No	10/ 42
Published protocol	Journal publication/ Trial registry only /Not reported	4/ 22/ 26

^a^Other includes: Anaesthesia, nephrology (2), neurology, diabetes care, musculoskeletal health (2)

Table 2 summarises aspects of the statistical methodology, with some emphasis on the stated (as opposed to actual) approach to potential early stopping for futility. Binary variables were used most frequently as the primary study outcome (63%), followed by continuous and time-to-event outcomes. The inter-quartile range of planned total sample sizes was from 133 to 586. Setting aside the four studies whose power could not be determined, all studies except one had planned power of at least 80%, and approximately 30% of trials had power exceeding 90%. This implies that most studies had a conventional (and reasonable) chance of detecting a treatment effect, assuming that the treatment effect size on which the power had been calculated was approximately correct.

**Table 2:** Statistical design features of 52 clinical trials that stopped for futility

Characteristics		N
Type of primary outcome	Binary/ Continuous/ time-to-event/ Trinomial	33/ 10/ 8/ 1
Planned sample size	Median (IQR)	209 (133–586)
Study power %	70–79 / 80–89 / 90 + / Unclear	1 / 32 / 15 / 4
Interim analyses planned?	Yes / No / Not stated	38 / 6 / 8
Number of interim analyses for futility planned	1 / 2 / 3 / Other ^a^ / Not stated	19 / 5 / 2 / 3 / 9
Basis for stopping rule	Not defined / Threshold / p-value / CP-related / Other	30 / 9 / 4 / 5 / 4
Information % at planned IA^b^	< 40 / 40–59 / 60–79 / Not stated or unclear / Other	7 / 17 / 9 / 19 / 4
Number of actual interim analyses	0 / 1 / 2 / Other^c^	2 / 41 / 7 / 2
Type of significance testing	One-sided / Two-sided / Unclear	14 / 33 / 5

*IA* Interim analysis

^a^: Other includes annual, at least annual, or indefinite

^b^: Distribution includes studies with stated information % for multiple IAs. “Other” includes studies defined by IAs planned at certain times or with a given number of patients

^c^: Other includes one study with 5 IA’s and one study with “annual” IA’s

We determined that 73% of the trials had planned at least one interim analysis for futility. In 6 (12%) trials, the interim analysis was not planned in advance, for a variety of reasons, such as: the analysis was carried out because a majority of patients were reporting no clinical benefit, despite some apparent improvement in the primary outcome variable; the trial’s DSMB requested an interim futility analysis because of funding difficulties; there was an interim analysis planned for possible treatment benefit, but conditional power was also computed to evaluate futility, because of a low patient recruitment rate; and the publication of disappointing results in another related study. In 15% of trials, it was not stated if the interim analysis was planned or unplanned. The most common number of planned interim analyses for futility, when stated, was 1 (45% of trials), but in most cases it was not clear if further interim analyses would have been planned after the actual stopping point of the study. However, in 19% of trials, the number of planned interim analyses was not pre-specified.

Table 2 also summarises the stated basis (if any) for the study’s futility stopping rule. More than half of the studies (58%) did not formally pre-specify a rule, guideline, or any other criteria for early stopping. Nine trials indicated that they would use a threshold value, either of a suitable test statistic, or as defined by the observed effect size. Four studies (8%) decided to use a *p*-value criterion, while one made a non-specific statement about potentially using some “futility” criterion. Only 5 RCT reports (10%) mentioned that they would consider conditional power. The criterion for one trial was to stop if the event rates in the two treatment groups showed the experimental to be doing worse than control.

Over one-third of trials did not state or were unclear about when their interim analyses would be carried out. Among those that did pre-specify this feature, about half of them planned an interim analysis when about 50% of the target total information would be available. Others planned an interim analysis as early as 25% or as late as 75%. Two trials planned interim analyses at specific times, such as annually or after a certain number of years.

Most trials (77%) had completed only one actual interim analysis before stopping for reasons of futility. However, there were two cases where, despite stating that the trial had been abandoned for futility, there was no actual interim analysis reported at all. The remaining studies had two or more interim analyses before stopping. About two-thirds of trials proposed to use two-sided significance tests, 27% with one-sided tests, and the remainder (8%) being unclear.

Table 3 describes the actual experience of stopping early for futility. In 58% of studies, the trial was stopped by the investigators, on advice from the DSMB to do so. A small number of studies (3, 6%) were – according to the trial publication - stopped by the DSMB alone. Many (29%) of studies did not report the decision-making process. The percentage of information available at the last interim analysis was quite variable, but as was the case for the planned interim analyses, the most common value was at or around the half-way point of the study. The earliest final interim analysis occurred at 11% information (2 studies), and the latest occurred in one study where the sample size for the interim analysis actually slightly exceeded the original target for the whole study. Among the studies where a comparison was possible, in almost every case the percentage of information available at the last interim analysis met the target for planned interim analyses (See Additional file 3 for details).

**Table 3:** Aspects of early stopping of 52 clinical trials that stopped for futility

Characteristics		N
Who made decision to stop early	Investigators on advice from DSMB / DSMB / Sponsor / Executive committee / Not stated	30 / 3 / 3 / 1 / 15
Actual information % at last IA	< 40 / 40–59 / 60–79 / 80+ / Not stated or unclear	11 / 20 / 12 / 6 / 3
Basis for stopping^a^	Continuation would not change results Futility mentioned Lack of treatment effect Conditional power Prior guidelines / boundary or threshold crossed “unlikely to become statistically significant”, or similar statement. Other	3 23 20 3 9 9 8
How futility was described	Futility claimed, no details Futility analysis described, but CP not reported CP reported in detail Not stated or unclear	35 4 11 2
Conditional power evaluation	Reported by authors / Calculated by us / Impossible to calculate	11 / 25 / 16
Analyses after last IA	Yes / No / Unclear or not applicable	21 / 23 / 6 / 2

^a^: Multiple responses possible

Various reasons were reported as the basis for stopping a trial early (Table 3). The most common reasons were that the desired treatment effect had not been observed, often with a verbal invocation of futility, in about 40% of trials. Many investigators indicated their general belief that the results would not change if the study were to continue, or that the final results would not be statistically significant, but without specifically mentioning the term “futility”. About 20% of studies stated that a pre-specified stopping boundary had been crossed, or prior guidelines were invoked. Other trials gave a variety of verbal summaries of the outcomes, such as stating that “outcomes did not differ” between treatment groups, that the data showed “insufficient efficacy”, or that the “intervention was inferior”. Some trials verbally described their conditional power, that it was “nearly zero”, or implied low conditional power by stating that the study had “almost no chance” or was “unlikely” to “achieve statistical significance”. One RCT claimed that there was an “impossibility of demonstrating efficacy”, while another described the results as showing “presumed” futility.

Two-thirds of studies made a claim of futility, but without providing any statistical details. Four studies indicated they had carried out a futility analysis, but did not show its results, while 11 studies gave explicit values of conditional power. Two of these 11 trials specified that their conditional power value had been calculated under an assumption that the current data trend would continue, while the others did not indicate their underlying assumption about future data. We were able to calculate conditional power ourselves for 25 studies, but for the 16 remaining studies, there was insufficient information available to calculate conditional power.

**Fig. 2:**
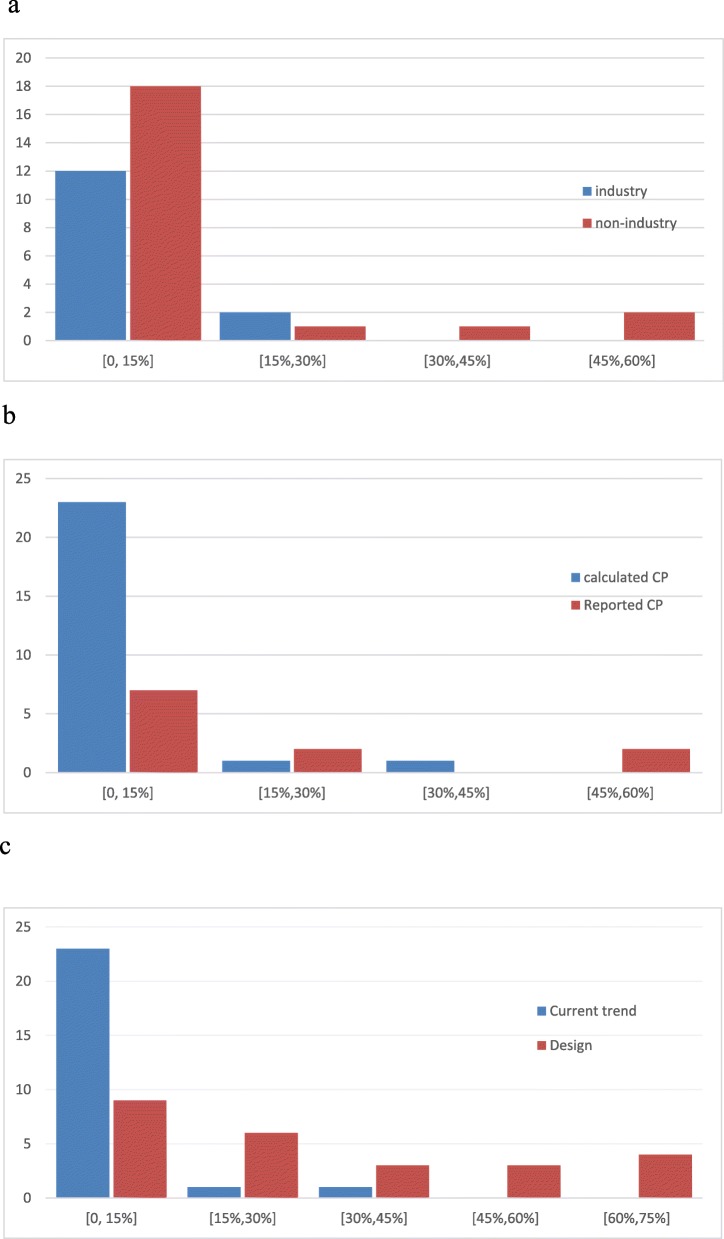
**a**: Distribution of conditional power by study sponsor: current trend assumption **b** Distribution of reported (11 trials) or calculated (25 trials) conditional power: current trend assumption. **c** Distribution of conditional power calculated by us (25 trials): current trend and design assumptions

We examined the distribution of conditional power for the trials where it could be obtained. Fig. 2 shows the distributions of conditional power by sponsor, according to whether it was reported by the investigators or calculated by us, or by which hypothesis was adopted for its calculation. Recall that, because an explicit statement of their assumptions was lacking in almost all studies where conditional power was reported by the authors, we treated all 11 of these trials as being under the current trend hypothesis. Whenever conditional power had been or could be calculated, it was typically low, especially under the current trend hypothesis. Fig. 2a indicates that low conditional power (say < 15%) was seen in most industry and non-industry trials, but there were a few non-industry trials where the conditional power was considerably higher.

Among the 25 trials whose conditional power was calculated by us, 23 had conditional power less than 15% under the current trend assumption (Fig. 2b). Eight of these trials had conditional power less than 15% under the original design assumption, 7 trials were in the 15–30%, 4 trials were in the range 30–50%, and 6 trials had relatively high conditional power (greater than 50%). Among the 11 trials with conditional power reported by investigators, 7 had conditional power less than 15% (one of them indicated that the current trend assumption was adopted, but the others did not specify what assumption had been used), two had conditional power larger than 15% (29 and 54%), and the remaining two studies reported their conditional power as less than 30% and less than 60% respectively, but without giving exact results.

Figure 2c shows the distribution of conditional power for the trials evaluated by us, under both the current trend and original design hypotheses. Not unexpectedly, conditional power is considerably higher with the design hypothesis, under which the pre-specified treatment effect size is retained. Many studies then have conditional power values which would probably argue in favour of continuing, based on conventional threshold values of conditional power (such as 10% or 15%). In contrast, the current trend hypothesis most often leads to conditional power values that do not meet such a threshold. From this comparison, we learn that the decision to stop for futility depends substantially on what assumption is made about the expected outcomes in future patients. Given that all our sampled trials did actually stop for futility, it appears that the decision may have been made more frequently on the basis of conditional power under the current trend model. Recall, however, conditional power or related ideas were indicated as a major reason for stopping in only a minority of studies.

There were 44 trials for which we could determine if additional patients had been included in the final reported analysis, but who had not contributed to the last interim analysis. In about half of them, data collection stopped immediately after the interim analysis, but in the others, patient follow-up and data collection continued, leading to a larger sample size at the final report. In one extreme case, the sample size in the final report was actually slightly larger than the original total target, despite stopping after an interim analysis. This suggests that, at least in some studies, executing an interim analysis for futility partially or completely failed in its objective of reducing its total sample size.

## Discussion

This paper has reviewed how interim analyses that led to an early stop for futility in clinical trials have been reported, in a wide variety of RCTs in the biomedical literature. Our survey of 52 trial reports showed that, from the perspective of conditional power, stopping early for futility was probably a reasonable decision in many cases, especially if the original design parameters are abandoned and the current data trend is believed to be accurate. However, if the design hypothesis is thought to have enduring credibility, conditional power then would often not be low enough to satisfy conventional thresholds for stopping the trial early for futility. Hence, the decision about stopping should necessarily involve some discussion about what may be expected in future data, if the trial were to continue.

On the one hand, if investigators believe that outcomes in future patients are accurately represented by the current data, and if conditional power is low at the interim analysis, that would be compatible with a recommendation to stop early. Relevant here are the findings by Rothwell et al. [11], who found that the observed effect sizes in a sample of trials were much smaller than their corresponding target effect sizes. On the other hand, if investigators feel that the original design and protocol remain valid, then they might conclude that the disappointing early results at the interim analysis are expected to be counterbalanced by more favourable experience in future patients; and in some of these cases, conditional power may be sufficiently high to support a decision to continue the study.

We found that documentation of the stopping rule or decision-making processes was often missing or vague. In many cases, it was unclear if the interim analysis had been planned in advance, or if other interim analyses might have occurred at a later date. There was often no pre-specification of how many interim analyses (if any) had been planned, or when they would take place. Although many trials claimed that futility was the basis for stopping a trial, there were few details about what specific criteria (if any) for futility had been applied. Many studies made only rather general or vague allusions to the concept of futility, but with no detailed analysis of the data in this regard. Disappointingly, only a few trials actually calculated conditional power, which can provide a more convincing demonstration of futility, or in other cases can support a decision to continue the study.

However, there were some trial reports where full details were reported: an example is the study by Powers et al. [S37], which shows their formal basis for potentially stopping at an interim analysis, the alternative hypotheses adopted when conditional power was calculated, the actual conditional power result on which stopping was based, and the fact that a pre-specified (and described) boundary for futility had been crossed.

Our findings agree closely with those of Avery et al. [12], whose focus is on internal pilot studies for clinical trials. They indicate that detailed reporting of the decision-making for stopping is uncommon*,* and they suggest that a pilot stage can provide a good opportunity to identify futile situations, and thus consider truncating a study. In related work, Sully et al. [13] simulated a sample of publicly funded trials in UK, and found that a single futility analysis with conditional power threshold of 30% could have correctly stopped 10 / 33 trials which in fact continued, but which ultimately showed negative results; such early stops would have saved many unnecessary patients from being recruited. Sully et al. conclude by suggesting that investigators should include interim analyses for futility in their study design, if possible. However, there may be a case for continuing the trial to completion, with the goal of narrowing the confidence interval on the treatment effect sufficiently to rule out any clinically relevant benefit. In more generality, CONSORT guidelines for adaptive designs are currently under development [14], which may lead to improved reporting practices; however, adequately reporting conditional power calculations is not a requirement of the current guidelines.

Most of the literature on interim analyses in clinical trials has focused on the inferential properties of early stopping rules, particularly the maintenance of the overall Type-I error rate with multiple interim analyses of the data, but relatively few authors have discussed the impact of early stopping rules on estimation of the treatment effect. We have previously [1] used the general theory of conditional power at the time of an interim analysis, and we were able to derive analytic expressions for several relevant parameters of interest, including: the mis-estimation of the treatment effect in studies that stop for reasons of futility; (ii) the estimation of treatment effect in studies that are completed, but only after an interim analysis that did not lead to an early stop for futility; (iii) the overall bias in the estimated treatment effect in a study with a futility stopping rule in its protocol; and (iv) the probability of actually stopping for futility at an interim analysis.

When these expressions were evaluated numerically for typical trial scenarios, we found that these parameters depend on various factors, including the magnitude of the underlying treatment effect, the treatment effect that the trial is designed to detect, planned study power, the number of planned interim analyses, and what assumptions are made about future data that might be observed after an interim analysis. In most practical situations, the probability of stopping early is small, and consequently the overall bias is also often small. Nevertheless, there is the potential for substantial underestimation of the treatment effect in studies that do actually stop for futility*.* We also illustrated these ideas using data from one clinical trial that did stop for futility after an interim analysis.

Schou and Marschener [15] have discussed the bias in estimating the treatment effect with a continuous outcome variable, calculated relative to the true effect; their theory pertains to trials with a stopping rule for benefit, but conditional on not actually stopping the trial. Their conditional relative bias was found to be approximately 10–20% for adequately powered studies that have one interim analysis at the halfway point, but it is larger if the interim analysis occurs later in the study. Schou and Marschener also consider meta-analyses, where they find that the inclusion of truncated studies is the preferred analytic strategy, because it leads to an “essentially unbiased” estimate of treatment effect.

Hughes et al. [16] showed by simulation that early stopping can either increase or decrease the strength of between-study heterogeneity in meta-analyses, and that this can affect later stages of the analysis. They indicate that because a single trial with a stopping rule would yield a biased estimate of the treatment effect, overviews including such trials would consequently also be biased; however, their numerical results suggest that the bias would be small as long as there are a reasonable number of studies available for the meta-analysis.

There are other concerns about potential bias in estimating the treatment effect in meta-analyses, over and above the general statistical effects that we considered in our earlier work. First, if there happens to be an early truncated trial, it may receive unduly large weight in any meta-analysis which is executed before other full studies are completed. This may lead to substantial mis-estimation of the treatment effect. Elsewhere, we have presented several real-world examples when such situations occurred, with negative consequences for patients [17]. We have also discussed an example of our meta-analysis of three trials of alternative ventilation strategies for patients with acute lung injuries; two of the trials were stopped for futility, and this prevented us from reaching a definitive conclusion about the preferred treatment [18].

Second, if the results of a truncated trial are accepted by the clinical community at face value, there may be a “freezing” effect, whereby the disappointing results from the truncated trial make it less likely that confirmatory studies on the same clinical question will actually take place. Furthermore, these effects may be accentuated by the relatively rapid publication of truncated studies (e.g. by editorial fast tracking of a paper), because of their apparently “definitive” results. We and others have discussed this issue in more detail elsewhere [1, 19].

While interim analyses are published quite frequently, under-reporting of this design feature exists, and therefore the actual use rate of stopping rules is greater than it appears in the published literature [2]; a particular problem is non-reporting of even the *existence* of a stopping rule for studies that actually complete their target total sample sizes, without the stopping rule being invoked. We acknowledge that there were only four trials with published protocols in our sample and that we did not contact trial investigators about unreported details of potentially existing stopping rules. A further issue is that a Data Monitoring Committee can over-rule the findings from an interim analysis, and they may recommend continuation of the study even though the early results (and the conditional power calculation in particular) are disappointing. Also, note that our survey included studies where the final sample size was larger than the sample size at the last interim analysis; again, the fact that an interim analysis had taken place may not be evident from the final study report for some studies.

## Conclusions

Although other factors will typically be involved in the decision to terminate a trial early, we conclude that, from the perspective of conditional power, stopping early for futility was probably reasonable in most of the trials we reviewed, as long as the interim data are representative of future data, if the study were to continue. In contrast, if one takes the position that the original design hypothesis, including the target effect size, are still valid, then conditional power may sometimes provide reasonable support for the trial to continue. However, documentation of the basis for stopping in our sample of trials was often missing or vague. Conditional power was not commonly calculated, and even in studies where it was calculated, the crucial assumptions concerning future data were often not specified.

Literature suggests that the inclusion of a stopping rule in a study protocol introduces the possibility of mis-estimation and/or bias in the treatment effect. Specifically, trials that do stop early for reasons of apparent futility will tend to under-estimate the underlying treatment effect; and studies with a protocol that specifies interim analyses, but which actually continue until the target total sample size is achieved, will be subject to overall bias in the estimated treatment effect. Accordingly, we believe it is important for trial reports to provide details of their plans for interim analyses. Reported estimates of treatment effects are more difficult to interpret if investigators fail to report aspects of their study design, such as if interim analyses (and how many) had been planned, when they would be executed, and what criteria would be applied to decide about stopping.

When studies do stop early, it is particularly important to know what the criteria had been applied for declaring futility, including what assumptions were made about future data, as these assumptions would importantly affect the value of conditional power, for example. If studies fail to provide the details of their interim analysis protocols, it becomes difficult to assess the impact of these aspects of the study design on their reported treatment effects. Interpretability of the results of truncated trials would be greatly enhanced by improved reporting of stopping rule protocols, and of their execution in practice.

The expected mis-estimation or bias in the treatment effect associated with futility stopping rules cannot easily be computed, because these quantities are functions of the true (and hence unknown) treatment effect itself. Nevertheless, general theory and simulation work can inform us about the general patterns of mis-estimation and bias to be anticipated for various typical scenarios [1]. Awareness of these tendencies should be helpful to investigators in the interpretation of trial results. At the very least, adequate reporting of interim analysis and stopping rule protocols should alert users of these results that trials that stop early for futility will yield under-estimates of treatment effects, possibly quite seriously.

In summary, our main findings are as follows:

- Many studies are unclear about if or when interim analyses would take place, and criteria for early stopping for futility are frequently not pre-specified.
- Many studies that stop early make general claims of futility, but without any formal analysis.
- The decision to stop for futility depends substantially on what assumption is made about the expected outcomes in future patients. The few studies that report conditional power calculations often do not state the critical assumption about expected outcomes in future data.
- Conditional power was typically low under the current trend assumption; however, under the original design hypothesis, the conditional power value would sometimes support continuation of the trial.
- Interpretation of trial results is made difficult by incomplete reporting of the plans for and execution of interim analyses for futility.

Based on these findings, we make the following recommendations:

- Pre-specified plans for interim analyses of futility should be reported; essential details include the number and timing of interim analyses, and criteria for declaring futility and stopping the trial.
- Conditional power should be calculated and reported at interim analyses, to support the decision to stop or continue a trial.
- The crucial assumption concerning the expected pattern of future data, on which basis conditional power is calculated, should be clearly stated and defended. Common choices are to assume that the current trend in the data will continue, or that the original design hypothesis will be retained.
- Investigators should be aware: 1) that trials that have stopped early for futility will generally under-estimate the treatment effect, possibly substantially; 2) that studies with a futility stopping rule, but which continue to completion will tend to over-estimate treatment benefit; and 3) that the inclusion of interim analyses for futility in a trial protocol will imply negative bias (over all studies, stopped or not) in the estimated treatment effect.

## Supplementary information

**Additional file 1: Table S1.** Summary of 52 randomised trials stopped early for futility: authors, subject, and futility analysis

**Additional file 2: Table S2.** Summary of 52 randomised trials stopped early for futility: selected statistical features

**Additional file 3: Table S3.** Summary of 52 randomised trials stopped early for futility: results

**Additional file 4.** References to sample of 52 stopped studies

## Data Availability

The data analysed during the current study is available in the manuscript and associated additional files.

## Abbreviations

DISCO: DISCOntinuation of RCTs
DSMB: Data and Safety Monitoring Board
