# Supplementary material for: A systematic survey of randomised trials that stopped early for reasons of futility
Source: BMC Med Res Methodol. 2020 Jan 16;20:10. doi: 10.1186/s12874-020-0899-1 (PMC6966801; doi:10.1186/s12874-020-0899-1)
Supplement: Supplementary file 4 — Additional file 4. References to sample of 52 stopped studies [file 12874_2020_899_MOESM4_ESM.docx]

**References to sample of 52 stopped studies**

S1. van Beekhuizen HJ, Tarimo V, Pembe AB, Fauteck H, Lotgering FK. A randomized controlled trial on the value of misoprostol for the treatment of retained placenta in a low-resource setting. Int J Gynaecol Obstet*.* 2013;122(3):234-237.

S2. Boggs DH, Robins HI, Langer CJ, Traynor AM, Berkowitz MJ, Mehta MP. Strategies to prevent brain metastasis in high-risk non-small-cell lung cancer: lessons learned from a randomized study of maintenance temozolomide versus observation. Clin Lung Cancer*.* 2014;15(6):433-440.

S3. Bolliger D, Seeberger MD, Lurati Buse GA, Christen P, Gurke L, Filipovic M. Randomized clinical trial of moxonidine in patients undergoing major vascular surgery. Br J Surg*.* 2007;94(12):1477-1484.

S4. Bottiger BW, Arntz HR, Chamberlain DA, et al. Thrombolysis during resuscitation for out-of-hospital cardiac arrest. N Engl J Med*.* 2008;359(25):2651-2662.

S5. Buchbinder SP, Mehrotra DV, Duerr A, et al. Efficacy assessment of a cell-mediated immunity HIV-1 vaccine (the Step Study): a double-blind, randomised, placebo-controlled, test-of-concept trial. Lancet*.* 2008;372(9653):1881-1893.

S6. Burgos J, Pijoan JI, Osuna C, et al. Increased pain relief with remifentanil does not improve the success rate of external cephalic version: a randomized controlled trial. Acta Obstet Gynecol Scand. 2016;95(5):547-554.

S7. Burnett AK, Hills RK, Hunter A, et al. The addition of arsenic trioxide to low-dose Ara-C in older patients with AML does not improve outcome. Leukemia*.* 2011;25(7):1122-1127.

S8. Carducci MA, Saad F, Abrahamsson PA, et al. A phase 3 randomized controlled trial of the efficacy and safety of atrasentan in men with metastatic hormone-refractory prostate cancer. Cancer*.* 2007;110(9):1959-1966.

S9. Carter TI, Fong ZV, Hyslop T, et al. A dual-institution randomized controlled trial of remnant closure after distal pancreatectomy: does the addition of a falciform patch and fibrin glue improve outcomes? Journal of Gastrointestinal Surgery. 2013;17(1):102-109.

S10. Casey EM, Harb W, Bradford D, et al. Randomized, double-blinded, multicenter, phase II study of pemetrexed, carboplatin, and bevacizumab with enzastaurin or placebo in chemonaive patients with stage IIIB/IV non-small cell lung cancer: Hoosier Oncology Group LUN06-116. J Thorac Oncol*.* 2010;5(11):1815-1820.

S11. Champlin RE, Perez WS, Passweg JR, et al. Bone marrow transplantation for severe aplastic anemia: a randomized controlled study of conditioning regimens. Blood*.* 2007;109(10):4582-4585.

S12. Clemons M, Joy AA, Abdulnabi R, et al. Phase II, double-blind, randomized trial of capecitabine plus enzastaurin versus capecitabine plus placebo in patients with metastatic or recurrent breast cancer after prior anthracycline and taxane therapy. Breast Cancer Res Treat. 2010;124(1):177-186.

S13. Combs CA, Garite TJ, Maurel K, et al. 17-hydroxyprogesterone caproate for preterm rupture of the membranes: a multicenter, randomized, double-blind, placebo-controlled trial. Am J Obstet Gynecol*.* 2015;213(3):364 e361-312.

S14. Dowson C, Sahai A, Watkins J, Dasgupta P, Khan MS. The safety and efficacy of botulinum toxin-A in the management of bladder oversensitivity: a randomised double-blind placebo-controlled trial. Int J Clin Pract*.* 2011;65(6):698-704.

S15. Erdmann E, Califf R, Gerstein HC, et al. Effects of the dual peroxisome proliferator-activated receptor activator aleglitazar in patients with Type 2 Diabetes mellitus or prediabetes. Am Heart J*.* 2015;170(1):117-122.

S16. Findling RL, Cavus I, Pappadopulos E, et al. Ziprasidone in adolescents with schizophrenia: results from a placebo-controlled efficacy and long-term open-extension study.J Child Adolesc Psychopharmacol*.* 2013;23(8):531-544.

S17. Fleishaker DL, Garcia Meijide JA, Petrov A, et al. Maraviroc, a chemokine receptor-5 antagonist, fails to demonstrate efficacy in the treatment of patients with rheumatoid arthritis in a randomized, double-blind placebo-controlled trial. Arthritis Res Ther*.* 2012;14(1):R11.

S18. Garcia-Manero G, Gartenberg G, Steensma DP, et al. A phase 2, randomized, double-blind, multicenter study comparing siltuximab plus best supportive care (BSC) with placebo plus BSC in anemic patients with International Prognostic Scoring System low- or intermediate-1-risk myelodysplastic syndrome. Am J Hematol*.* 2014;89(9):E156-162.

S19. Gilron I, Tu D, Dumerton-Shore D, et al. The effect of triple vs. double nonopioid therapy on postoperative pain and functional outcome after abdominal hysterectomy: a randomised double-blind control trial. Eur J Anaesthesiol*.* 2015;32(4):269-276.

S20/21. Haas SK, Freund M, Heigener D, et al. Low-molecular-weight heparin versus placebo for the prevention of venous thromboembolism in metastatic breast cancer or stage III/IV lung cancer. Clin Appl Thromb Hemost*.* 2012;18(2):159-165.

S22. Haik S, Marcon G, Mallet A, et al. Doxycycline in Creutzfeldt-Jakob disease: a phase 2, randomised, double-blind, placebo-controlled trial. Lancet Neurol*.* 2014;13(2):150-158.

S23. Hennerici MG, Kay R, Bogousslavsky J, et al. Intravenous ancrod for acute ischaemic stroke in the European Stroke Treatment with Ancrod Trial: a randomised controlled trial. Lancet*.* 2006;368(9550):1871-1878.

S24. Hogg RJ, Bay RC, Jennette JC, et al. Randomized controlled trial of mycophenolate mofetil in children, adolescents, and adults with IgA nephropathy. Am J Kidney Dis*.* 2015;66(5):783-791.

S25/26. Hudson M, Greenbaum A, Brenton L, et al. Adjunctive transcutaneous ultrasound with thrombolysis: results of the PLUS (Perfusion by ThromboLytic and UltraSound) trial. JACC Cardiovasc Interv*.* 2010;3(3):352-359.

S27. Jalal SI, Riggs HD, Melnyk A, et al. Updated survival and outcomes for older adults with inoperable stage III non-small-cell lung cancer treated with cisplatin, etoposide, and concurrent chest radiation with or without consolidation docetaxel: analysis of a phase III trial from the Hoosier Oncology Group (HOG) and US Oncology. Ann Oncol. 2012;23(7):1730-1738.

S28. Juthani-Mehta M, Van Ness PH, McGloin J, et al. A cluster-randomized controlled trial of a multicomponent intervention protocol for pneumonia prevention among nursing home elders. Clin Infect Dis*.* 2015;60(6):849-857.

S29. Karthaus M, Ballo H, Abenhardt W, et al. Prospective, double-blind, placebo-controlled, multicenter, randomized phase III study with orally administered budesonide for prevention of irinotecan (conditional powerT-11)-induced diarrhea in patients with advanced colorectal cancer. Oncology*.* 2005;68(4-6):326-332.

S30. Kummar S, Oza AM, Fleming GF, et al. Randomized trial of oral cyclophosphamide (C) with or without veliparib (V), an oral poly (ADP-ribose) polymerase (PARP) inhibitor, in patients with recurrent BRCA-positive ovarian, or primary peritoneal or high-grade serous ovarian carcinoma. Journal of Clinical Oncology*.* 2012;30(15_suppl 2012: 5020).

S31. Levenick JM, Gordon SR, Fadden LL, et al. Rectal Indomethacin Does Not Prevent Post-ERconditional power Pancreatitis in Consecutive Patients. Gastroenterology. 2016;150(4):911-917; quiz e919.

S32. Moreau P, Hullin C, Garban F, et al. Tandem autologous stem cell transplantation in high-risk de novo multiple myeloma: final results of the prospective and randomized IFM 99-04 protocol. Blood. 2006;107(1):397-403.

S33. Nicholls SJ, Kastelein JJ, Schwartz GG, et al. Varespladib and cardiovascular events in patients with an acute coronary syndrome: the VISTA-16 randomized clinical trial. JAMA. 2014;311(3):252-262.

S34. Opal S, Laterre PF, Abraham E, et al. Recombinant human platelet-activating factor acetylhydrolase for treatment of severe sepsis: results of a phase III, multicenter, randomized, double-blind, placebo-controlled, clinical trial. Crit Care Med. 2004;32(2):332-341.

S35. Park LT, Lener MS, Hopkins M, et al. A Double-Blind, Placebo-Controlled, Pilot Study of Riluzole Monotherapy for Acute Bipolar Depression. J Clin Psychopharmacol*.* 2017;37(3):355-358.

S36. Philipp T, Martinez F, Geiger H, et al. Candesartan improves blood pressure control and reduces proteinuria in renal transplant recipients: results from SECRET. Nephrol Dial Transplant. 2010;25(3):967-976.

S37. Powers WJ, Clarke WR, Grubb RL, Jr., et al. Extracranial-intracranial bypass surgery for stroke prevention in hemodynamic cerebral ischemia: the Carotid Occlusion Surgery Study randomized trial. JAMA. 2011;306(18):1983-1992.

S38. Powles T, Wheater M, Din O, et al. A Randomised Phase 2 Study of AZD2014 Versus Everolimus in Patients with VEGF-Refractory Metastatic Clear Cell Renal Cancer. Eur Urol. 2016;69(3):450-456.

S39. Rees CM, Eaton S, Kiely EM, Wade AM, McHugh K, Pierro A. Peritoneal drainage or laparotomy for neonatal bowel perforation? A randomized controlled trial. Ann Surg. 2008;248(1):44-51.

S40. Roehr B. HIV prevention trial in women is abandoned after drugs show no impact on infection rates. BMJ. 2011;342:d2613.

S41. Sacktor N, Miyahara S, Deng L, et al. Minocycline treatment for HIV-associated cognitive impairment: results from a randomized trial. Neurology. 2011;77(12):1135-1142.

S42. Schadendorf D, Ugurel S, Schuler-Thurner B, et al. Dacarbazine (DTIC) versus vaccination with autologous peptide-pulsed dendritic cells (DC) in first-line treatment of patients with metastatic melanoma: a randomized phase III trial of the DC study group of the DeCOG. Ann Oncol. 2006;17(4):563-570.

S43. Schnabel D, Grasemann C, Staab D, Wollmann H, Ratjen F, German Cystic Fibrosis Growth Hormone Study G. A multicenter, randomized, double-blind, placebo-controlled trial to evaluate the metabolic and respiratory effects of growth hormone in children with cystic fibrosis. Pediatrics. 2007;119(6):e1230-1238.

S44. Schumacher M, Schmidt D, Jurklies B, et al. Central retinal artery occlusion: local intra-arterial fibrinolysis versus conservative treatment, a multicenter randomized trial. Ophthalmology. 2010;117(7):1367-1375 e1361.

S45. Simons KS, Laheij RJF, van den Boogaard M, et al. Dynamic light application therapy to reduce the incidence and duration of delirium in intensive-care patients: a randomised controlled trial. The Lancet Respiratory Medicine. 2016;4(3):194-202.

S46. Smolen JS, Weinblatt ME, van der Heijde D, et al. Efficacy and safety of tabalumab, an anti-B-cell-activating factor monoclonal antibody, in patients with rheumatoid arthritis who had an inadequate response to methotrexate therapy: results from a phase III multicentre, randomised, double-blind study. Ann Rheum Dis. 2015;74(8):1567-1570.

S47/48. Strasser F, Luftner D, Possinger K, et al. Comparison of orally administered cannabis extract and delta-9-tetrahydrocannabinol in treating patients with cancer-related anorexia-cachexia syndrome: a multicenter, phase III, randomized, double-blind, placebo-controlled clinical trial from the Cannabis-In-Cachexia-Study-Group. J Clin Oncol. 2006;24(21):3394-3400.

S49. Tilley BC, Mainous AG, 3rd, Elm JJ, et al. A randomized recruitment intervention trial in Parkinson's disease to increase participant diversity: early stopping for lack of efficacy. Clin Trials. 2012;9(2):188-197.

S50. Torgano G, Zecca B, Monzani V, et al. Effect of intravenous tirofiban and aspirin in reducing short-term and long-term neurologic deficit in patients with ischemic stroke: a double-blind randomized trial. Cerebrovasc Dis. 2010;29(3):275-281.

S51. Wadhwa A, Kabon B, Fleischmann E, Kurz A, Sessler DI. Supplemental postoperative oxygen does not reduce surgical site infection and major healing-related complications from bariatric surgery in morbidly obese patients: a randomized, blinded trial. Anesth Analg. 2014;119(2):357-365.

S52. Willson DF, Thomas NJ, Tamburro R, et al. Pediatric calfactant in acute respiratory distress syndrome trial. Pediatr Crit Care Med. 2013;14(7):657-665.
